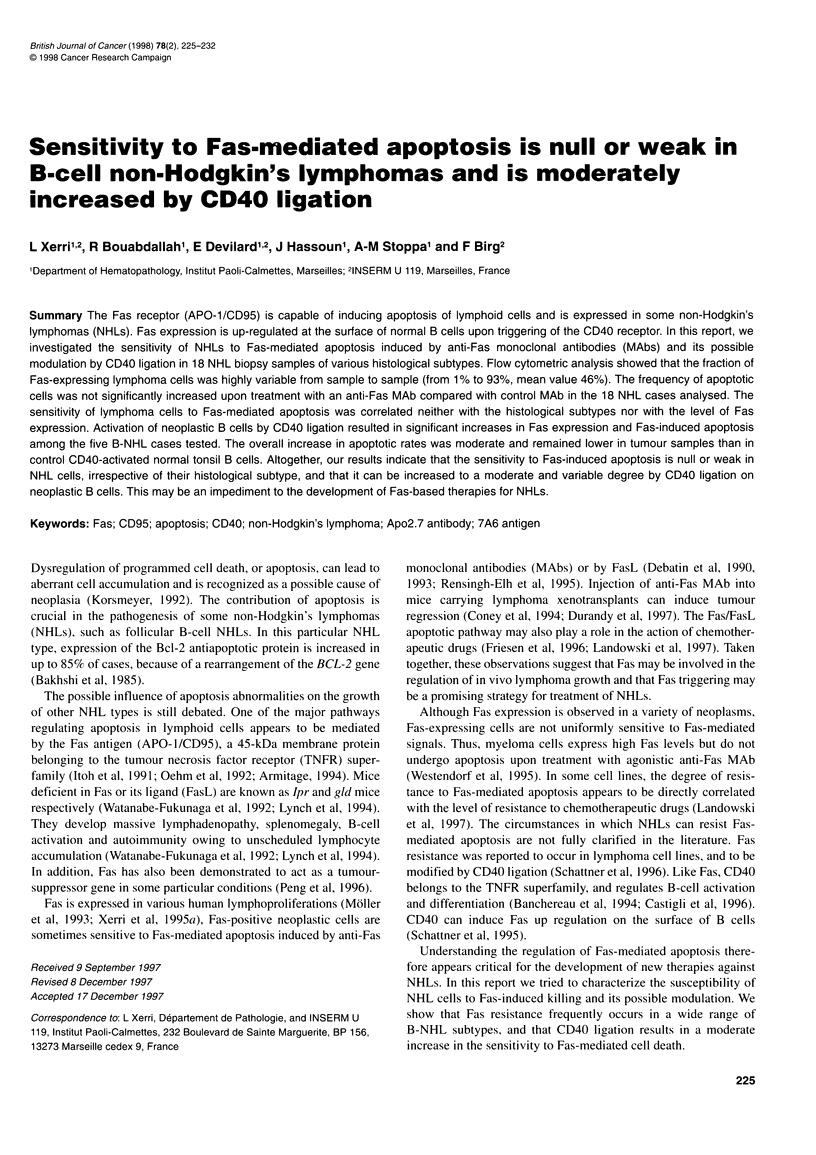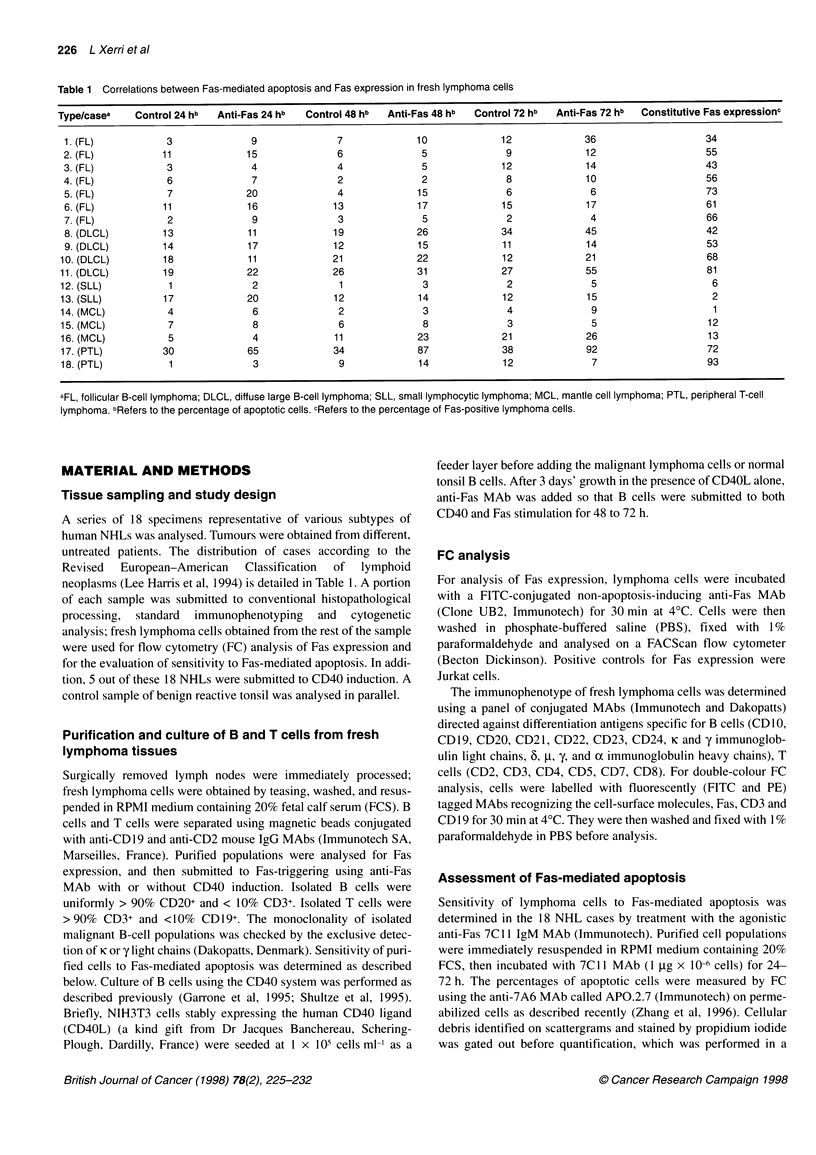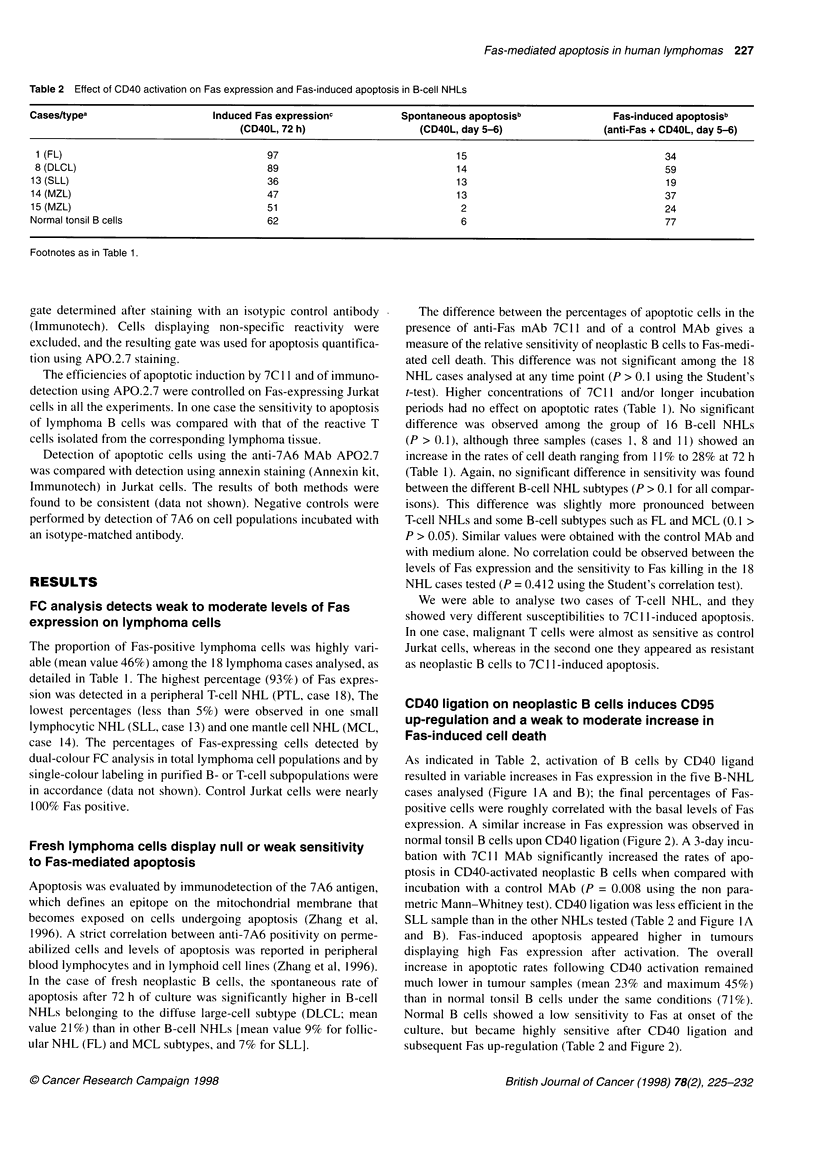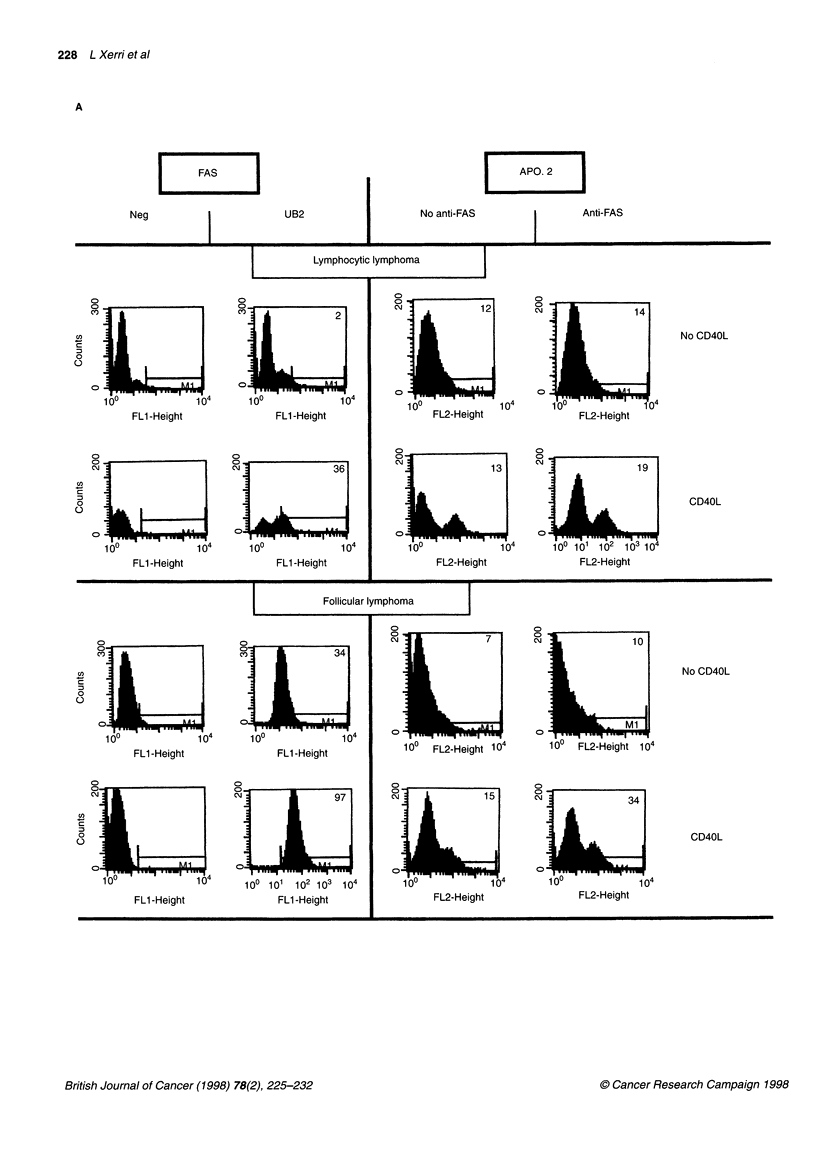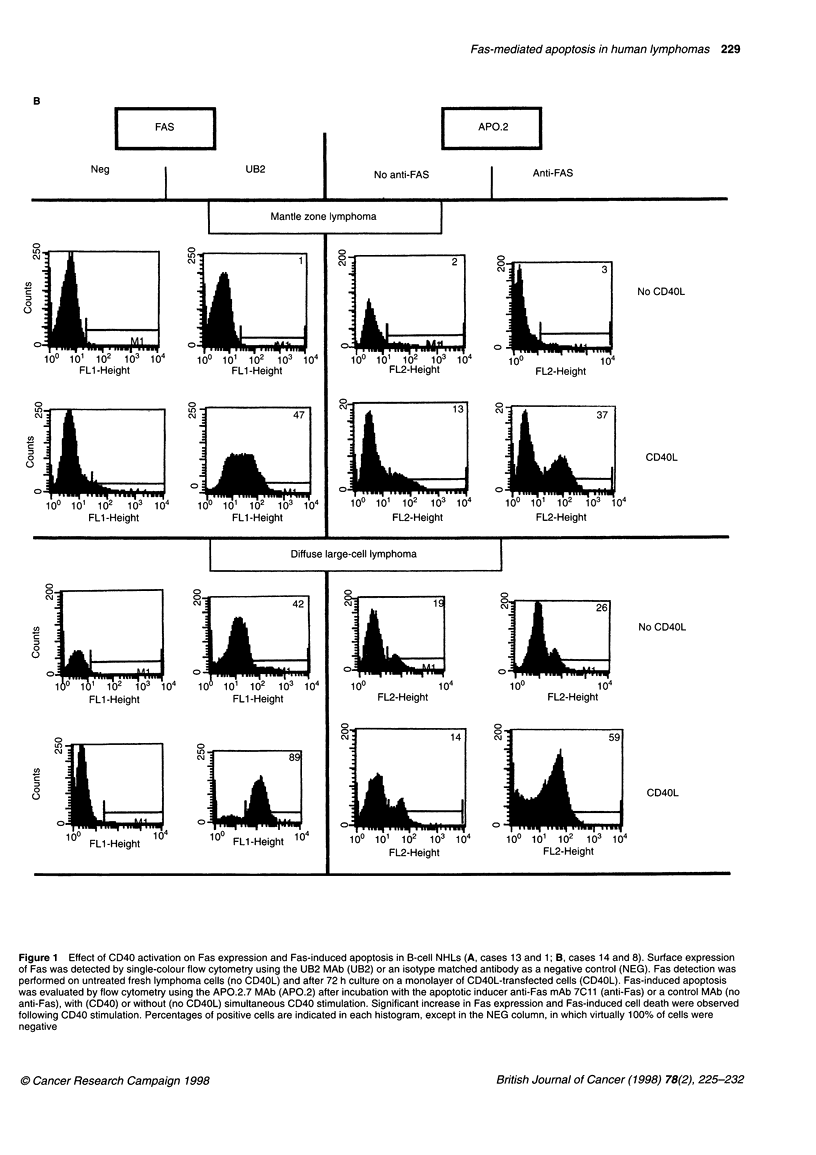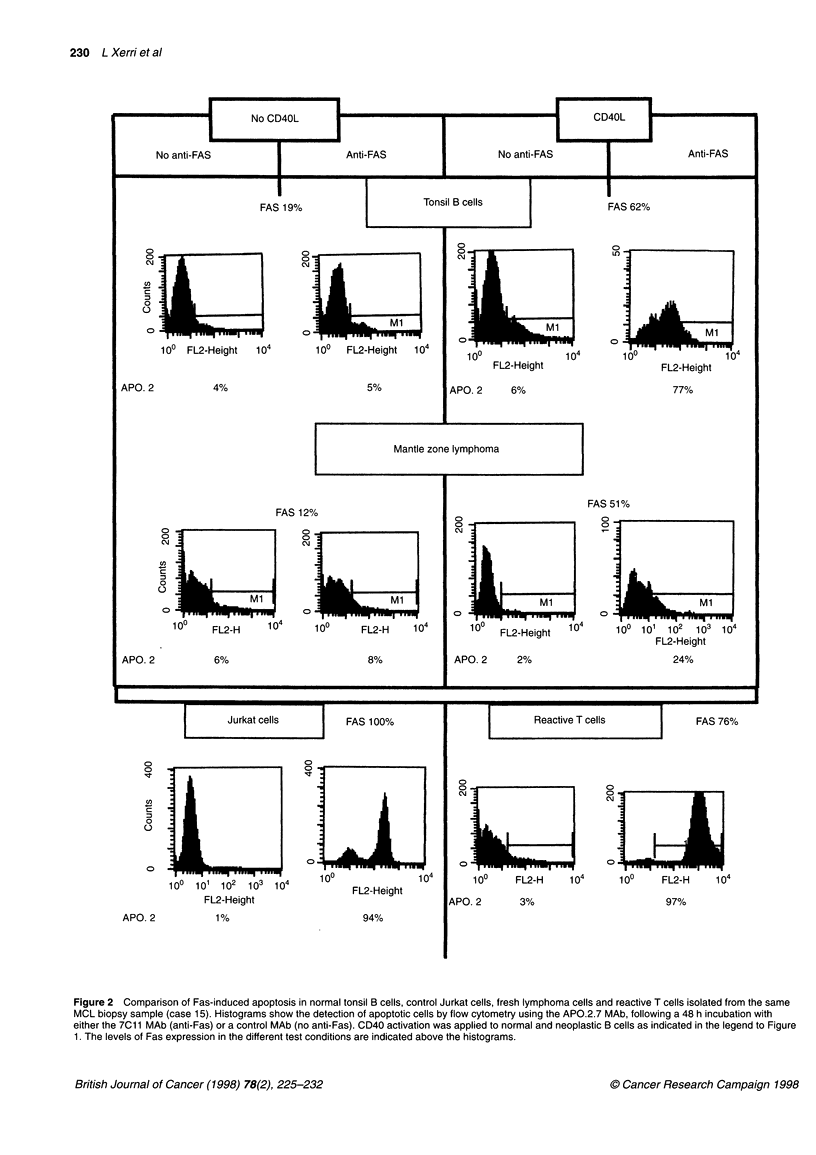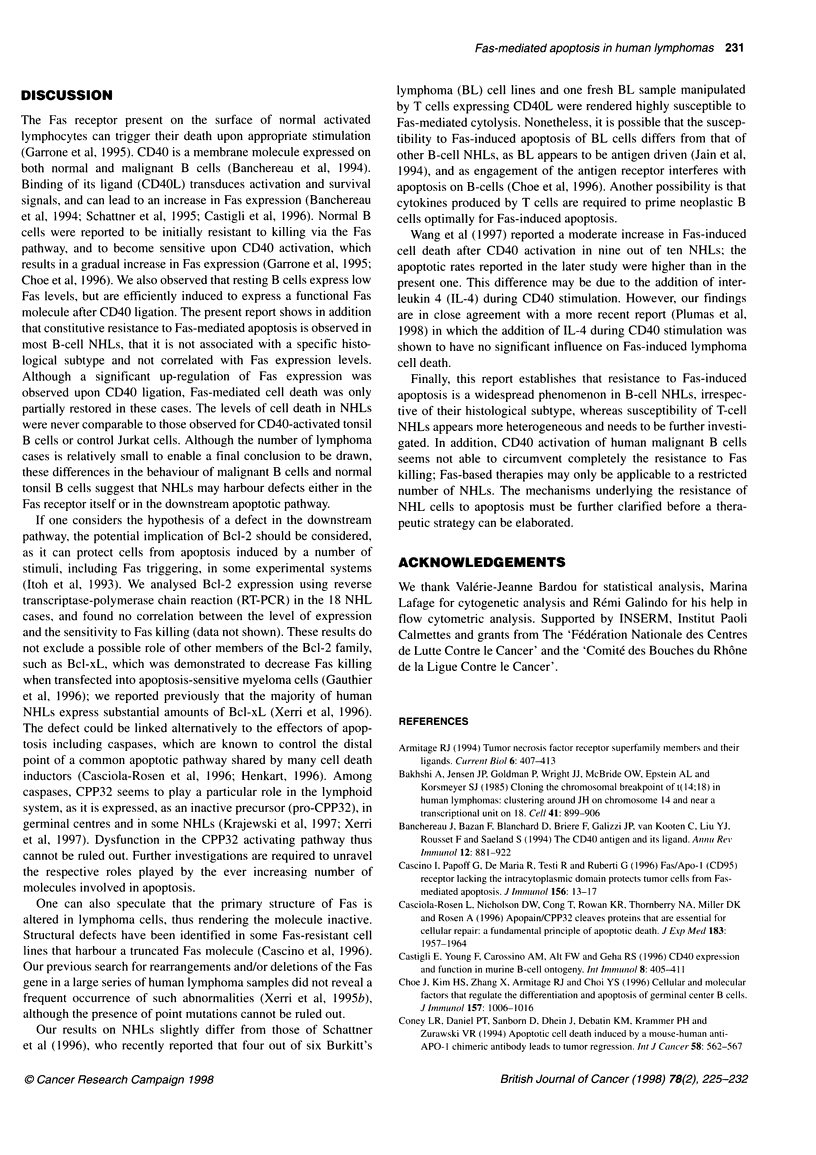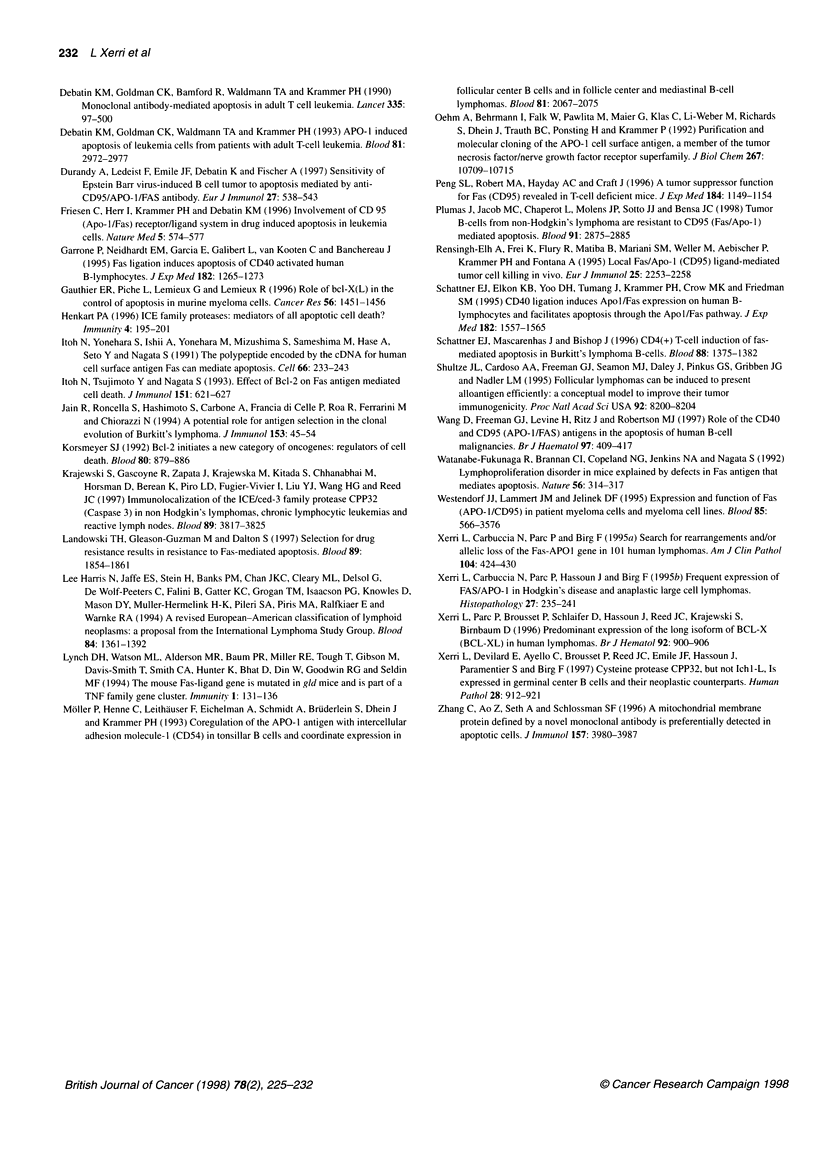# Sensitivity to Fas-mediated apoptosis is null or weak in B-cell non-Hodgkin's lymphomas and is moderately increased by CD40 ligation.

**DOI:** 10.1038/bjc.1998.469

**Published:** 1998-07

**Authors:** L. Xerri, R. Bouabdallah, E. Devilard, J. Hassoun, A. M. Stoppa, F. Birg

**Affiliations:** Department of Hematopathology, Institut Paoli-Calmettes, Marseilles, France.

## Abstract

The Fas receptor (APO-1/CD95) is capable of inducing apoptosis of lymphoid cells and is expressed in some non-Hodgkin's lymphomas (NHLs). Fas expression is up-regulated at the surface of normal B cells upon triggering of the CD40 receptor. In this report, we investigated the sensitivity of NHLs to Fas-mediated apoptosis induced by anti-Fas monoclonal antibodies (MAbs) and its possible modulation by CD40 ligation in 18 NHL biopsy samples of various histological subtypes. Flow cytometric analysis showed that the fraction of Fas-expressing lymphoma cells was highly variable from sample to sample (from 1% to 93%, mean value 46%). The frequency of apoptotic cells was not significantly increased upon treatment with an anti-Fas MAb compared with control MAb in the 18 NHL cases analysed. The sensitivity of lymphoma cells to Fas-mediated apoptosis was correlated neither with the histological subtypes nor with the level of Fas expression. Activation of neoplastic B cells by CD40 ligation resulted in significant increases in Fas expression and Fas-induced apoptosis among the five B-NHL cases tested. The overall increase in apoptotic rates was moderate and remained lower in tumour samples than in control CD40-activated normal tonsil B cells. Altogether, our results indicate that the sensitivity to Fas-induced apoptosis is null or weak in NHL cells, irrespective of their histological subtype, and that it can be increased to a moderate and variable degree by CD40 ligation on neoplastic B cells. This may be an impediment to the development of Fas-based therapies for NHLs.


British Journal of Cancer (1998) 78(2), 225–232
© 1998 Cancer Research Campaign

### Sensitivity to Fas-mediated apoptosis is null or weak in B-cell non-Hodgkin's lymphomas and is moderately increased by CD40 ligation

L Xerri[1,2], R Bouabdallah[1], E Devilard[1,2], J Hassoun[1], A-M Stoppa[1] and F Birg[2]

[1]Department of Hematopathology, Institut Paoli-Calmettes, Marseilles; [2]INSERM U 119, Marseilles, France

**Summary** The Fas receptor (APO-1/CD95) is capable of inducing apoptosis of lymphoid cells and is expressed in some non-Hodgkin's lymphomas (NHLs). Fas expression is up-regulated at the surface of normal B cells upon triggering of the CD40 receptor. In this report, we investigated the sensitivity of NHLs to Fas-mediated apoptosis induced by anti-Fas monoclonal antibodies (MAbs) and its possible modulation by CD40 ligation in 18 NHL biopsy samples of various histological subtypes. Flow cytometric analysis showed that the fraction of Fas-expressing lymphoma cells was highly variable from sample to sample (from 1% to 93%, mean value 46%). The frequency of apoptotic cells was not significantly increased upon treatment with an anti-Fas MAb compared with control MAb in the 18 NHL cases analysed. The sensitivity of lymphoma cells to Fas-mediated apoptosis was correlated neither with the histological subtypes nor with the level of Fas expression. Activation of neoplastic B cells by CD40 ligation resulted in significant increases in Fas expression and Fas-induced apoptosis among the five B-NHL cases tested. The overall increase in apoptotic rates was moderate and remained lower in tumour samples than in control CD40-activated normal tonsil B cells. Altogether, our results indicate that the sensitivity to Fas-induced apoptosis is null or weak in NHL cells, irrespective of their histological subtype, and that it can be increased to a moderate and variable degree by CD40 ligation on neoplastic B cells. This may be an impediment to the development of Fas-based therapies for NHLs.

**Keywords:** Fas; CD95; apoptosis; CD40; non-Hodgkin's lymphoma; Apo2.7 antibody; 7A6 antigen

Dysregulation of programmed cell death, or apoptosis, can lead to aberrant cell accumulation and is recognized as a possible cause of neoplasia (Korsmeyer, 1992). The contribution of apoptosis is crucial in the pathogenesis of some non-Hodgkin's lymphomas (NHLs), such as follicular B-cell NHLs. In this particular NHL type, expression of the Bcl-2 antiapoptotic protein is increased in up to 85% of cases, because of a rearrangement of the *BCL-2* gene (Bakhshi et al, 1985).

The possible influence of apoptosis abnormalities on the growth of other NHL types is still debated. One of the major pathways regulating apoptosis in lymphoid cells appears to be mediated by the Fas antigen (APO-1/CD95), a 45-kDa membrane protein belonging to the tumour necrosis factor receptor (TNFR) superfamily (Itoh et al, 1991; Oehm et al, 1992; Armitage, 1994). Mice deficient in Fas or its ligand (FasL) are known as *lpr* and *gld* mice respectively (Watanabe-Fukunaga et al, 1992; Lynch et al, 1994). They develop massive lymphadenopathy, splenomegaly, B-cell activation and autoimmunity owing to unscheduled lymphocyte accumulation (Watanabe-Fukunaga et al, 1992; Lynch et al, 1994). In addition, Fas has also been demonstrated to act as a tumour-suppressor gene in some particular conditions (Peng et al, 1996).

Fas is expressed in various human lymphoproliferations (Möller et al, 1993; Xerri et al, 1995*a*), Fas-positive neoplastic cells are sometimes sensitive to Fas-mediated apoptosis induced by anti-Fas monoclonal antibodies (MAbs) or by FasL (Debatin et al, 1990, 1993; Rensingh-Elh et al, 1995). Injection of anti-Fas MAb into mice carrying lymphoma xenotransplants can induce tumour regression (Coney et al, 1994; Durandy et al, 1997). The Fas/FasL apoptotic pathway may also play a role in the action of chemotherapeutic drugs (Friesen et al, 1996; Landowski et al, 1997). Taken together, these observations suggest that Fas may be involved in the regulation of in vivo lymphoma growth and that Fas triggering may be a promising strategy for treatment of NHLs.

Although Fas expression is observed in a variety of neoplasms, Fas-expressing cells are not uniformly sensitive to Fas-mediated signals. Thus, myeloma cells express high Fas levels but do not undergo apoptosis upon treatment with agonistic anti-Fas MAb (Westendorf et al, 1995). In some cell lines, the degree of resistance to Fas-mediated apoptosis appears to be directly correlated with the level of resistance to chemotherapeutic drugs (Landowski et al, 1997). The circumstances in which NHLs can resist Fas-mediated apoptosis are not fully clarified in the literature. Fas resistance was reported to occur in lymphoma cell lines, and to be modified by CD40 ligation (Schattner et al, 1996). Like Fas, CD40 belongs to the TNFR superfamily, and regulates B-cell activation and differentiation (Banchereau et al, 1994; Castigli et al, 1996). CD40 can induce Fas up regulation on the surface of B cells (Schattner et al, 1995).

Understanding the regulation of Fas-mediated apoptosis therefore appears critical for the development of new therapies against NHLs. In this report we tried to characterize the susceptibility of NHL cells to Fas-induced killing and its possible modulation. We show that Fas resistance frequently occurs in a wide range of B-NHL subtypes, and that CD40 ligation results in a moderate increase in the sensitivity to Fas-mediated cell death.

*Received 9 September 1997*
*Revised 8 December 1997*
*Accepted 17 December 1997*

*Correspondence to*: L Xerri, Département de Pathologie, and INSERM U 119, Institut Paoli-Calmettes, 232 Boulevard de Sainte Marguerite, BP 156, 13273 Marseille cedex 9, France

**Table 1** Correlations between Fas-mediated apoptosis and Fas expression in fresh lymphoma cells

| Type/case[a] | Control 24 h[b] | Anti-Fas 24 h[b] | Control 48 h[b] | Anti-Fas 48 h[b] | Control 72 h[b] | Anti-Fas 72 h[b] | Constitutive Fas expression[c] |
|---|---|---|---|---|---|---|---|
| 1. (FL) | 3 | 9 | 7 | 10 | 12 | 36 | 34 |
| 2. (FL) | 11 | 15 | 6 | 5 | 9 | 12 | 55 |
| 3. (FL) | 3 | 4 | 4 | 5 | 12 | 14 | 43 |
| 4. (FL) | 6 | 7 | 2 | 2 | 8 | 10 | 56 |
| 5. (FL) | 7 | 20 | 4 | 15 | 6 | 6 | 73 |
| 6. (FL) | 11 | 16 | 13 | 17 | 15 | 17 | 61 |
| 7. (FL) | 2 | 9 | 3 | 5 | 2 | 4 | 66 |
| 8. (DLCL) | 13 | 11 | 19 | 26 | 34 | 45 | 42 |
| 9. (DLCL) | 14 | 17 | 12 | 15 | 11 | 14 | 53 |
| 10. (DLCL) | 18 | 11 | 21 | 22 | 12 | 21 | 68 |
| 11. (DLCL) | 19 | 22 | 26 | 31 | 27 | 55 | 81 |
| 12. (SLL) | 1 | 2 | 1 | 3 | 2 | 5 | 6 |
| 13. (SLL) | 17 | 20 | 12 | 14 | 12 | 15 | 2 |
| 14. (MCL) | 4 | 6 | 2 | 3 | 4 | 9 | 1 |
| 15. (MCL) | 7 | 8 | 6 | 8 | 3 | 5 | 12 |
| 16. (MCL) | 5 | 4 | 11 | 23 | 21 | 26 | 13 |
| 17. (PTL) | 30 | 65 | 34 | 87 | 38 | 92 | 72 |
| 18. (PTL) | 1 | 3 | 9 | 14 | 12 | 7 | 93 |

[a]FL, follicular B-cell lymphoma; DLCL, diffuse large B-cell lymphoma; SLL, small lymphocytic lymphoma; MCL, mantle cell lymphoma; PTL, peripheral T-cell lymphoma. [b]Refers to the percentage of apoptotic cells. [c]Refers to the percentage of Fas-positive lymphoma cells.

### MATERIAL AND METHODS

### Tissue sampling and study design

A series of 18 specimens representative of various subtypes of human NHLs was analysed. Tumours were obtained from different, untreated patients. The distribution of cases according to the Revised European–American Classification of lymphoid neoplasms (Lee Harris et al, 1994) is detailed in Table 1. A portion of each sample was submitted to conventional histopathological processing, standard immunophenotyping and cytogenetic analysis; fresh lymphoma cells obtained from the rest of the sample were used for flow cytometry (FC) analysis of Fas expression and for the evaluation of sensitivity to Fas-mediated apoptosis. In addition, 5 out of these 18 NHLs were submitted to CD40 induction. A control sample of benign reactive tonsil was analysed in parallel.

### Purification and culture of B and T cells from fresh lymphoma tissues

Surgically removed lymph nodes were immediately processed; fresh lymphoma cells were obtained by teasing, washed, and resuspended in RPMI medium containing 20% fetal calf serum (FCS). B cells and T cells were separated using magnetic beads conjugated with anti-CD19 and anti-CD2 mouse IgG MAbs (Immunotech SA, Marseilles, France). Purified populations were analysed for Fas expression, and then submitted to Fas-triggering using anti-Fas MAb with or without CD40 induction. Isolated B cells were uniformly > 90% $CD20^+$ and < 10% $CD3^+$. Isolated T cells were > 90% $CD3^+$ and <10% $CD19^+$. The monoclonality of isolated malignant B-cell populations was checked by the exclusive detection of κ or γ light chains (Dakopatts, Denmark). Sensitivity of purified cells to Fas-mediated apoptosis was determined as described below. Culture of B cells using the CD40 system was performed as described previously (Garrone et al, 1995; Shultze et al, 1995). Briefly, NIH3T3 cells stably expressing the human CD40 ligand (CD40L) (a kind gift from Dr Jacques Banchereau, Schering-Plough, Dardilly, France) were seeded at $1 \times 10^5$ cells $ml^{-1}$ as a feeder layer before adding the malignant lymphoma cells or normal tonsil B cells. After 3 days' growth in the presence of CD40L alone, anti-Fas MAb was added so that B cells were submitted to both CD40 and Fas stimulation for 48 to 72 h.

### FC analysis

For analysis of Fas expression, lymphoma cells were incubated with a FITC-conjugated non-apoptosis-inducing anti-Fas MAb (Clone UB2, Immunotech) for 30 min at 4°C. Cells were then washed in phosphate-buffered saline (PBS), fixed with 1% paraformaldehyde and analysed on a FACScan flow cytometer (Becton Dickinson). Positive controls for Fas expression were Jurkat cells.

The immunophenotype of fresh lymphoma cells was determined using a panel of conjugated MAbs (Immunotech and Dakopatts) directed against differentiation antigens specific for B cells (CD10, CD19, CD20, CD21, CD22, CD23, CD24, κ and γ immunoglobulin light chains, δ, μ, γ, and α immunoglobulin heavy chains), T cells (CD2, CD3, CD4, CD5, CD7, CD8). For double-colour FC analysis, cells were labelled with fluorescently (FITC and PE) tagged MAbs recognizing the cell-surface molecules, Fas, CD3 and CD19 for 30 min at 4°C. They were then washed and fixed with 1% paraformaldehyde in PBS before analysis.

### Assessment of Fas-mediated apoptosis

Sensitivity of lymphoma cells to Fas-mediated apoptosis was determined in the 18 NHL cases by treatment with the agonistic anti-Fas 7C11 IgM MAb (Immunotech). Purified cell populations were immediately resuspended in RPMI medium containing 20% FCS, then incubated with 7C11 MAb (1 μg × $10^{-6}$ cells) for 24–72 h. The percentages of apoptotic cells were measured by FC using the anti-7A6 MAb called APO.2.7 (Immunotech) on permeabilized cells as described recently (Zhang et al, 1996). Cellular debris identified on scattergrams and stained by propidium iodide was gated out before quantification, which was performed in a

 © Cancer Research Campaign 1998

**Table 2** Effect of CD40 activation on Fas expression and Fas-induced apoptosis in B-cell NHLs

| Cases/type[a] | Induced Fas expression[c] (CD40L, 72 h) | Spontaneous apoptosis[b] (CD40L, day 5–6) | Fas-induced apoptosis[b] (anti-Fas + CD40L, day 5–6) |
|---|---|---|---|
| 1 (FL) | 97 | 15 | 34 |
| 8 (DLCL) | 89 | 14 | 59 |
| 13 (SLL) | 36 | 13 | 19 |
| 14 (MZL) | 47 | 13 | 37 |
| 15 (MZL) | 51 | 2 | 24 |
| Normal tonsil B cells | 62 | 6 | 77 |

Footnotes as in Table 1.

gate determined after staining with an isotypic control antibody (Immunotech). Cells displaying non-specific reactivity were excluded, and the resulting gate was used for apoptosis quantification using APO.2.7 staining.

The efficiencies of apoptotic induction by 7C11 and of immunodetection using APO.2.7 were controlled on Fas-expressing Jurkat cells in all the experiments. In one case the sensitivity to apoptosis of lymphoma B cells was compared with that of the reactive T cells isolated from the corresponding lymphoma tissue.

Detection of apoptotic cells using the anti-7A6 MAb APO2.7 was compared with detection using annexin staining (Annexin kit, Immunotech) in Jurkat cells. The results of both methods were found to be consistent (data not shown). Negative controls were performed by detection of 7A6 on cell populations incubated with an isotype-matched antibody.

### RESULTS

### FC analysis detects weak to moderate levels of Fas expression on lymphoma cells

The proportion of Fas-positive lymphoma cells was highly variable (mean value 46%) among the 18 lymphoma cases analysed, as detailed in Table 1. The highest percentage (93%) of Fas expression was detected in a peripheral T-cell NHL (PTL, case 18), The lowest percentages (less than 5%) were observed in one small lymphocytic NHL (SLL, case 13) and one mantle cell NHL (MCL, case 14). The percentages of Fas-expressing cells detected by dual-colour FC analysis in total lymphoma cell populations and by single-colour labeling in purified B- or T-cell subpopulations were in accordance (data not shown). Control Jurkat cells were nearly 100% Fas positive.

### Fresh lymphoma cells display null or weak sensitivity to Fas-mediated apoptosis

Apoptosis was evaluated by immunodetection of the 7A6 antigen, which defines an epitope on the mitochondrial membrane that becomes exposed on cells undergoing apoptosis (Zhang et al, 1996). A strict correlation between anti-7A6 positivity on permeabilized cells and levels of apoptosis was reported in peripheral blood lymphocytes and in lymphoid cell lines (Zhang et al, 1996). In the case of fresh neoplastic B cells, the spontaneous rate of apoptosis after 72 h of culture was significantly higher in B-cell NHLs belonging to the diffuse large-cell subtype (DLCL; mean value 21%) than in other B-cell NHLs [mean value 9% for follicular NHL (FL) and MCL subtypes, and 7% for SLL].

The difference between the percentages of apoptotic cells in the presence of anti-Fas mAb 7C11 and of a control MAb gives a measure of the relative sensitivity of neoplastic B cells to Fas-mediated cell death. This difference was not significant among the 18 NHL cases analysed at any time point ($P > 0.1$ using the Student's *t*-test). Higher concentrations of 7C11 and/or longer incubation periods had no effect on apoptotic rates (Table 1). No significant difference was observed among the group of 16 B-cell NHLs ($P > 0.1$), although three samples (cases 1, 8 and 11) showed an increase in the rates of cell death ranging from 11% to 28% at 72 h (Table 1). Again, no significant difference in sensitivity was found between the different B-cell NHL subtypes ($P > 0.1$ for all comparisons). This difference was slightly more pronounced between T-cell NHLs and some B-cell subtypes such as FL and MCL ($0.1 > P > 0.05$). Similar values were obtained with the control MAb and with medium alone. No correlation could be observed between the levels of Fas expression and the sensitivity to Fas killing in the 18 NHL cases tested ($P = 0.412$ using the Student's correlation test).

We were able to analyse two cases of T-cell NHL, and they showed very different susceptibilities to 7C11-induced apoptosis. In one case, malignant T cells were almost as sensitive as control Jurkat cells, whereas in the second one they appeared as resistant as neoplastic B cells to 7C11-induced apoptosis.

### CD40 ligation on neoplastic B cells induces CD95 up-regulation and a weak to moderate increase in Fas-induced cell death

As indicated in Table 2, activation of B cells by CD40 ligand resulted in variable increases in Fas expression in the five B-NHL cases analysed (Figure 1A and B); the final percentages of Fas-positive cells were roughly correlated with the basal levels of Fas expression. A similar increase in Fas expression was observed in normal tonsil B cells upon CD40 ligation (Figure 2). A 3-day incubation with 7C11 MAb significantly increased the rates of apoptosis in CD40-activated neoplastic B cells when compared with incubation with a control MAb ($P = 0.008$ using the non parametric Mann–Whitney test). CD40 ligation was less efficient in the SLL sample than in the other NHLs tested (Table 2 and Figure 1A and B). Fas-induced apoptosis appeared higher in tumours displaying high Fas expression after activation. The overall increase in apoptotic rates following CD40 activation remained much lower in tumour samples (mean 23% and maximum 45%) than in normal tonsil B cells under the same conditions (71%). Normal B cells showed a low sensitivity to Fas at onset of the culture, but became highly sensitive after CD40 ligation and subsequent Fas up-regulation (Table 2 and Figure 2).

© *Cancer Research Campaign 1998*

*© Cancer Research Campaign 1998*

**Figure 1** Effect of CD40 activation on Fas expression and Fas-induced apoptosis in B-cell NHLs (**A**, cases 13 and 1; **B**, cases 14 and 8). Surface expression of Fas was detected by single-colour flow cytometry using the UB2 MAb (UB2) or an isotype matched antibody as a negative control (NEG). Fas detection was performed on untreated fresh lymphoma cells (no CD40L) and after 72 h culture on a monolayer of CD40L-transfected cells (CD40L). Fas-induced apoptosis was evaluated by flow cytometry using the APO.2.7 MAb (APO.2) after incubation with the apoptotic inducer anti-Fas mAb 7C11 (anti-Fas) or a control MAb (no anti-Fas), with (CD40) or without (no CD40L) simultaneous CD40 stimulation. Significant increase in Fas expression and Fas-induced cell death were observed following CD40 stimulation. Percentages of positive cells are indicated in each histogram, except in the NEG column, in which virtually 100% of cells were negative

*© Cancer Research Campaign 1998*

Figure 2 Comparison of Fas-induced apoptosis in normal tonsil B cells, control Jurkat cells, fresh lymphoma cells and reactive T cells isolated from the same MCL biopsy sample (case 15). Histograms show the detection of apoptotic cells by flow cytometry using the APO.2.7 MAb, following a 48 h incubation with either the 7C11 MAb (anti-Fas) or a control MAb (no anti-Fas). CD40 activation was applied to normal and neoplastic B cells as indicated in the legend to Figure 1. The levels of Fas expression in the different test conditions are indicated above the histograms.

### DISCUSSION

The Fas receptor present on the surface of normal activated lymphocytes can trigger their death upon appropriate stimulation (Garrone et al, 1995). CD40 is a membrane molecule expressed on both normal and malignant B cells (Banchereau et al, 1994). Binding of its ligand (CD40L) transduces activation and survival signals, and can lead to an increase in Fas expression (Banchereau et al, 1994; Schattner et al, 1995; Castigli et al, 1996). Normal B cells were reported to be initially resistant to killing via the Fas pathway, and to become sensitive upon CD40 activation, which results in a gradual increase in Fas expression (Garrone et al, 1995; Choe et al, 1996). We also observed that resting B cells express low Fas levels, but are efficiently induced to express a functional Fas molecule after CD40 ligation. The present report shows in addition that constitutive resistance to Fas-mediated apoptosis is observed in most B-cell NHLs, that it is not associated with a specific histological subtype and not correlated with Fas expression levels. Although a significant up-regulation of Fas expression was observed upon CD40 ligation, Fas-mediated cell death was only partially restored in these cases. The levels of cell death in NHLs were never comparable to those observed for CD40-activated tonsil B cells or control Jurkat cells. Although the number of lymphoma cases is relatively small to enable a final conclusion to be drawn, these differences in the behaviour of malignant B cells and normal tonsil B cells suggest that NHLs may harbour defects either in the Fas receptor itself or in the downstream apoptotic pathway.

If one considers the hypothesis of a defect in the downstream pathway, the potential implication of Bcl-2 should be considered, as it can protect cells from apoptosis induced by a number of stimuli, including Fas triggering, in some experimental systems (Itoh et al, 1993). We analysed Bcl-2 expression using reverse transcriptase-polymerase chain reaction (RT-PCR) in the 18 NHL cases, and found no correlation between the level of expression and the sensitivity to Fas killing (data not shown). These results do not exclude a possible role of other members of the Bcl-2 family, such as Bcl-xL, which was demonstrated to decrease Fas killing when transfected into apoptosis-sensitive myeloma cells (Gauthier et al, 1996); we reported previously that the majority of human NHLs express substantial amounts of Bcl-xL (Xerri et al, 1996). The defect could be linked alternatively to the effectors of apoptosis including caspases, which are known to control the distal point of a common apoptotic pathway shared by many cell death inductors (Casciola-Rosen et al, 1996; Henkart, 1996). Among caspases, CPP32 seems to play a particular role in the lymphoid system, as it is expressed, as an inactive precursor (pro-CPP32), in germinal centres and in some NHLs (Krajewski et al, 1997; Xerri et al, 1997). Dysfunction in the CPP32 activating pathway thus cannot be ruled out. Further investigations are required to unravel the respective roles played by the ever increasing number of molecules involved in apoptosis.

One can also speculate that the primary structure of Fas is altered in lymphoma cells, thus rendering the molecule inactive. Structural defects have been identified in some Fas-resistant cell lines that harbour a truncated Fas molecule (Cascino et al, 1996). Our previous search for rearrangements and/or deletions of the Fas gene in a large series of human lymphoma samples did not reveal a frequent occurrence of such abnormalities (Xerri et al, 1995*b*), although the presence of point mutations cannot be ruled out.

Our results on NHLs slightly differ from those of Schattner et al (1996), who recently reported that four out of six Burkitt's lymphoma (BL) cell lines and one fresh BL sample manipulated by T cells expressing CD40L were rendered highly susceptible to Fas-mediated cytolysis. Nonetheless, it is possible that the susceptibility to Fas-induced apoptosis of BL cells differs from that of other B-cell NHLs, as BL appears to be antigen driven (Jain et al, 1994), and as engagement of the antigen receptor interferes with apoptosis on B-cells (Choe et al, 1996). Another possibility is that cytokines produced by T cells are required to prime neoplastic B cells optimally for Fas-induced apoptosis.

Wang et al (1997) reported a moderate increase in Fas-induced cell death after CD40 activation in nine out of ten NHLs; the apoptotic rates reported in the later study were higher than in the present one. This difference may be due to the addition of interleukin 4 (IL-4) during CD40 stimulation. However, our findings are in close agreement with a more recent report (Plumas et al, 1998) in which the addition of IL-4 during CD40 stimulation was shown to have no significant influence on Fas-induced lymphoma cell death.

Finally, this report establishes that resistance to Fas-induced apoptosis is a widespread phenomenon in B-cell NHLs, irrespective of their histological subtype, whereas susceptibility of T-cell NHLs appears more heterogeneous and needs to be further investigated. In addition, CD40 activation of human malignant B cells seems not able to circumvent completely the resistance to Fas killing; Fas-based therapies may only be applicable to a restricted number of NHLs. The mechanisms underlying the resistance of NHL cells to apoptosis must be further clarified before a therapeutic strategy can be elaborated.

### ACKNOWLEDGEMENTS

We thank Valérie-Jeanne Bardou for statistical analysis, Marina Lafage for cytogenetic analysis and Rémi Galindo for his help in flow cytometric analysis. Supported by INSERM, Institut Paoli Calmettes and grants from The 'Fédération Nationale des Centres de Lutte Contre le Cancer' and the 'Comité des Bouches du Rhône de la Ligue Contre le Cancer'.

### REFERENCES

Armitage RJ (1994) Tumor necrosis factor receptor superfamily members and their ligands. *Current Biol* **6**: 407–413

Bakhshi A, Jensen JP, Goldman P, Wright JJ, McBride OW, Epstein AL and Korsmeyer SJ (1985) Cloning the chromosomal breakpoint of t(14;18) in human lymphomas: clustering around JH on chromosome 14 and near a transcriptional unit on 18. *Cell* **41**: 899–906

Banchereau J, Bazan F, Blanchard D, Briere F, Galizzi JP, van Kooten C, Liu YJ, Rousset F and Saeland S (1994) The CD40 antigen and its ligand. *Annu Rev Immunol* **12**: 881–922

Cascino I, Papoff G, De Maria R, Testi R and Ruberti G (1996) Fas/Apo-1 (CD95) receptor lacking the intracytoplasmic domain protects tumor cells from Fas-mediated apoptosis. *J Immunol* **156**: 13–17

Casciola-Rosen L, Nicholson DW, Cong T, Rowan KR, Thornberry NA, Miller DK and Rosen A (1996) Apopain/CPP32 cleaves proteins that are essential for cellular repair: a fundamental principle of apoptotic death. *J Exp Med* **183**: 1957–1964

Castigli E, Young F, Carossino AM, Alt FW and Geha RS (1996) CD40 expression and function in murine B-cell ontogeny. *Int Immunol* **8**: 405–411

Choe J, Kim HS, Zhang X, Armitage RJ and Choi YS (1996) Cellular and molecular factors that regulate the differentiation and apoptosis of germinal center B cells. *J Immunol* **157**: 1006–1016

Coney LR, Daniel PT, Sanborn D, Dhein J, Debatin KM, Krammer PH and Zurawski VR (1994) Apoptotic cell death induced by a mouse-human anti-APO-1 chimeric antibody leads to tumor regression. *Int J Cancer* **58**: 562–567

© Cancer Research Campaign 1998

Debatin KM, Goldman CK, Bamford R, Waldmann TA and Krammer PH (1990) Monoclonal antibody-mediated apoptosis in adult T cell leukemia. *Lancet* **335**: 97–500

Debatin KM, Goldman CK, Waldmann TA and Krammer PH (1993) APO-1 induced apoptosis of leukemia cells from patients with adult T-cell leukemia. *Blood* **81**: 2972–2977

Durandy A, Ledeist F, Emile JF, Debatin K and Fischer A (1997) Sensitivity of Epstein Barr virus-induced B cell tumor to apoptosis mediated by anti-CD95/APO-1/FAS antibody. *Eur J Immunol* **27**: 538–543

Friesen C, Herr I, Krammer PH and Debatin KM (1996) Involvement of CD 95 (Apo-1/Fas) receptor/ligand system in drug induced apoptosis in leukemia cells. *Nature Med* **5**: 574–577

Garrone P, Neidhardt EM, Garcia E, Galibert L, van Kooten C and Banchereau J (1995) Fas ligation induces apoptosis of CD40 activated human B-lymphocytes. *J Exp Med* **182**: 1265–1273

Gauthier ER, Piche L, Lemieux G and Lemieux R (1996) Role of bcl-X(L) in the control of apoptosis in murine myeloma cells. *Cancer Res* **56**: 1451–1456

Henkart PA (1996) ICE family proteases: mediators of all apoptotic cell death? *Immunity* **4**: 195–201

Itoh N, Yonehara S, Ishii A, Yonehara M, Mizushima S, Sameshima M, Hase A, Seto Y and Nagata S (1991) The polypeptide encoded by the cDNA for human cell surface antigen Fas can mediate apoptosis. *Cell* **66**: 233–243

Itoh N, Tsujimoto Y and Nagata S (1993). Effect of Bcl-2 on Fas antigen mediated cell death. *J Immunol* **151**: 621–627

Jain R, Roncella S, Hashimoto S, Carbone A, Francia di Celle P, Roa R, Ferrarini M and Chiorazzi N (1994) A potential role for antigen selection in the clonal evolution of Burkitt's lymphoma. *J Immunol* **153**: 45–54

Korsmeyer SJ (1992) Bcl-2 initiates a new category of oncogenes: regulators of cell death. *Blood* **80**: 879–886

Krajewski S, Gascoyne R, Zapata J, Krajewska M, Kitada S, Chhanabhai M, Horsman D, Berean K, Piro LD, Fugier-Vivier I, Liu YJ, Wang HG and Reed JC (1997) Immunolocalization of the ICE/ced-3 family protease CPP32 (Caspase 3) in non Hodgkin's lymphomas, chronic lymphocytic leukemias and reactive lymph nodes. *Blood* **89**: 3817–3825

Landowski TH, Gleason-Guzman M and Dalton S (1997) Selection for drug resistance results in resistance to Fas-mediated apoptosis. *Blood* **89**: 1854–1861

Lee Harris N, Jaffe ES, Stein H, Banks PM, Chan JKC, Cleary ML, Delsol G, De Wolf-Peeters C, Falini B, Gatter KC, Grogan TM, Isaacson PG, Knowles D, Mason DY, Muller-Hermelink H-K, Pileri SA, Piris MA, Ralfkiaer E and Warnke RA (1994) A revised European–American classification of lymphoid neoplasms: a proposal from the International Lymphoma Study Group. *Blood* **84**: 1361–1392

Lynch DH, Watson ML, Alderson MR, Baum PR, Miller RE, Tough T, Gibson M, Davis-Smith T, Smith CA, Hunter K, Bhat D, Din W, Goodwin RG and Seldin MF (1994) The mouse Fas-ligand gene is mutated in *gld* mice and is part of a TNF family gene cluster. *Immunity* **1**: 131–136

Möller P, Henne C, Leithäuser F, Eichelman A, Schmidt A, Brüderlein S, Dhein J and Krammer PH (1993) Coregulation of the APO-1 antigen with intercellular adhesion molecule-1 (CD54) in tonsillar B cells and coordinate expression in follicular center B cells and in follicle center and mediastinal B-cell lymphomas. *Blood* **81**: 2067–2075

Oehm A, Behrmann I, Falk W, Pawlita M, Maier G, Klas C, Li-Weber M, Richards S, Dhein J, Trauth BC, Ponsting H and Krammer P (1992) Purification and molecular cloning of the APO-1 cell surface antigen, a member of the tumor necrosis factor/nerve growth factor receptor superfamily. *J Biol Chem* **267**: 10709–10715

Peng SL, Robert MA, Hayday AC and Craft J (1996) A tumor suppressor function for Fas (CD95) revealed in T-cell deficient mice. *J Exp Med* **184**: 1149–1154

Plumas J, Jacob MC, Chaperot L, Molens JP, Sotto JJ and Bensa JC (1998) Tumor B-cells from non-Hodgkin's lymphoma are resistant to CD95 (Fas/Apo-1) mediated apoptosis. *Blood* **91**: 2875–2885

Rensingh-Elh A, Frei K, Flury R, Matiba B, Mariani SM, Weller M, Aebischer P, Krammer PH and Fontana A (1995) Local Fas/Apo-1 (CD95) ligand-mediated tumor cell killing in vivo. *Eur J Immunol* **25**: 2253–2258

Schattner EJ, Elkon KB, Yoo DH, Tumang J, Krammer PH, Crow MK and Friedman SM (1995) CD40 ligation induces Apo1/Fas expression on human B-lymphocytes and facilitates apoptosis through the Apo1/Fas pathway. *J Exp Med* **182**: 1557–1565

Schattner EJ, Mascarenhas J and Bishop J (1996) CD4(+) T-cell induction of fas-mediated apoptosis in Burkitt's lymphoma B-cells. *Blood* **88**: 1375–1382

Shultze JL, Cardoso AA, Freeman GJ, Seamon MJ, Daley J, Pinkus GS, Gribben JG and Nadler LM (1995) Follicular lymphomas can be induced to present alloantigen efficiently: a conceptual model to improve their tumor immunogenicity. *Proc Natl Acad Sci* USA **92**: 8200–8204

Wang D, Freeman GJ, Levine H, Ritz J and Robertson MJ (1997) Role of the CD40 and CD95 (APO-1/FAS) antigens in the apoptosis of human B-cell malignancies. *Br J Haematol* **97**: 409–417

Watanabe-Fukunaga R, Brannan CI, Copeland NG, Jenkins NA and Nagata S (1992) Lymphoproliferation disorder in mice explained by defects in Fas antigen that mediates apoptosis. *Nature* **56**: 314–317

Westendorf JJ, Lammert JM and Jelinek DF (1995) Expression and function of Fas (APO-1/CD95) in patient myeloma cells and myeloma cell lines. *Blood* **85**: 566–3576

Xerri L, Carbuccia N, Parc P and Birg F (1995*a*) Search for rearrangements and/or allelic loss of the Fas-APO1 gene in 101 human lymphomas. *Am J Clin Pathol* **104**: 424–430

Xerri L, Carbuccia N, Parc P, Hassoun J and Birg F (1995*b*) Frequent expression of FAS/APO-1 in Hodgkin's disease and anaplastic large cell lymphomas. *Histopathology* **27**: 235–241

Xerri L, Parc P, Brousset P, Schlaifer D, Hassoun J, Reed JC, Krajewski S, Birnbaum D (1996) Predominant expression of the long isoform of BCL-X (BCL-XL) in human lymphomas. *Br J Hematol* **92**: 900–906

Xerri L, Devilard E, Ayello C, Brousset P, Reed JC, Emile JF, Hassoun J, Paramentier S and Birg F (1997) Cysteine protease CPP32, but not Ich1-L, Is expressed in germinal center B cells and their neoplastic counterparts. *Human Pathol* **28**: 912–921

Zhang C, Ao Z, Seth A and Schlossman SF (1996) A mitochondrial membrane protein defined by a novel monoclonal antibody is preferentially detected in apoptotic cells. *J Immunol* **157**: 3980–3987

© Cancer Research Campaign 1998